# Biometry, Refractive Errors, and the Results of Cataract Surgery: A Large Sample Study

**DOI:** 10.1155/2021/9918763

**Published:** 2021-04-27

**Authors:** Iván Hernández-López, Sahily Estradé-Fernández, Taimí Cárdenas-Díaz, Alfo José Batista-Leyva

**Affiliations:** ^1^Instituto Cubano de Oftalmología “Ramón Pando Ferrer”, La Habana 11500, Cuba; ^2^Departamento de Física Atómica y Molecular, Instituto Superior de Tecnologías y Ciencias Aplicadas, Universidad de la Habana, La Habana 10400, Cuba

## Abstract

The statistical characteristics of biometry and refractive error in a large sample of cataractous Cuban patients are presented, comparing between sexes and age groups. All patients were studied at the Cuban Institute of Ophthalmology “Ramón Pando Ferrer,” La Habana. The sample consists of 28252 eyes of 25068 patients, subjected to cataract surgery during the time period between 2006 and 2019. Their biometry was obtained using IOL Master devices; also, visual acuity, refraction, and corneal power were registered. After surgery, the visual acuity and refraction were measured. The refractive prediction error was determined. For patients with both eyes registered, anisometropia was also calculated. Age and sex were used to segment the data. The preoperative biometric parameters show highly significant differences between sexes, with male eyes being longer and with a deeper anterior chamber but with a thinner lens. Also, keratometry shows highly significant differences, with female eyes being steeper than male. Before surgery, both sexes have myopic eyes as average, with males being more myopic than females (*p* < 0.001). After surgery, the average spherical equivalent is −0.36 D and female eyes are more myopic than males (*p* < 0.001). Visual acuity with and without distance correction has a significant increase after surgery. These results are of importance not only for Cuba but also for other countries with a large Cuban population and/or similar ethnic composition, such as the USA (particularly the south of Florida), Spain, and many countries in Latin America.

## 1. Introduction

Latest advances in surgical techniques and intraocular lens designs have notably improved the postoperative refractive results of modern cataract surgery, increasing patients' expectations of good visual outcome without the use of spectacles. This outcome depends on the accurate prediction of the power of the implanted intraocular lens (IOL), which in turn depends mainly on preoperative biometry data [1]. Optical biometers have become the gold standard for ocular biometry, resulting in eye measurements being more reliable and allowing the evaluation of new parameters [2]. IOL Master 700, based on swept source optical coherence tomography (SS-OCT), is more effective in obtaining biometric measurements in eyes with posterior subcapsular and dense nuclear cataracts than its predecessor series, based on partial coherence interferometry (PCI). However, an excellent agreement has been demonstrated between these two optical technologies, regarding axial length, anterior chamber depth, and steepest and flattest keratometric values [3]. In a recent comparative study, it was found that, despite IOLMaster 700 having a superior acquisition rate than IOLMaster 500, both devices showed good agreement, and found statistical differences were clinically negligible [4]. Meanwhile, knowing ocular biometric characteristics of a population is an important factor to be considered in designing intraocular lenses and improving IOL calculation methods for selecting the most appropriate lenses for patients [5, 6]. The relation of ocular biometry with diabetic retinopathy has been reviewed, highlighting the importance of further biometric studies to elucidate the influence of high myopia and ocular biometry in the protective mechanisms of the eye [7]. Hence, several studies describing ocular biometric characteristics of different populations have been conducted across the world [6, 7]. In an extensive review, the prevalence of myopia in populations of different continents is studied, finding larger number of myopic eyes in Asian populations than in other regions of the world [8].

Several studies deal with the differences among genders in the biometric parameters. In Refs. [9, 10], authors found significantly longer eyes and deeper anterior chambers in men than in women in British and Chinese adult populations, respectively. These findings are consistent with the results of a Latino study [11]. A recent review [6] found this trend in a number of different studies. Regarding average corneal power, differences associated with sex vary among studies. In Ref. [12], studying an African population, authors did not find differences between sexes in astigmatism. The results of different studies are difficult to compare, due to the differences among them (ethnicity of the sample, sample size, measurement method). All these factors highlight the importance of studies associated with a given population.

Published studies of ocular biometric parameters in Cuban population are scarce and their casuistic have included no more than three hundred eyes [13–16]. To our knowledge, there has not been published before a large sample study of biometric values for the Cuban population. “Ramon Pando Ferrer” Cuban Institute of Ophthalmology is a national reference center, where thousands of patients from all island provinces are admitted for cataract surgery every year. For that reason, it was chosen as the setting by the present research work in order to provide the largest and more representative hospital-based population study of ocular biometric characteristics and refractive visual outcomes of cataract patients in Cuba. The results have importance not only to Cuba but also to other countries with a large Cuban population or similar ethnic composition.

## 2. Materials and Methods

A retrospective study of 28252 eyes (female 17811, male 10441) of 25068 patients (female 15745, male 9323), who underwent uneventful cataract surgery from January 2006 to December 2019, was performed at the “Ramón Pando Ferrer” Cuban Institute of Ophthalmology in Havana, Cuba. This study was approved by Institutional Scientific and Ethical Committees from the Ramón Pando Ferrer Cuban Institute of Ophthalmology and the Higher Institute of Technology and Applied Sciences (InSTEC, Spanish acronym), University of Havana, and conformed to the tenets of the Declaration of Helsinki.

Preoperative ocular biometric parameters, including axial length (AL), anterior chamber depth (ACD), lens thickness (LT), and corneal power (steepest keratometry SK, flattest keratometry FK, and average keratometry Kav), using optical biometers (IOL Master 3.0.2, 500 or 700, version 1.14, Carl Zeiss Meditec AG, Jena, Germany), were recorded, as well as visual acuity (uncorrected distance UDVA1 and corrected distance CDVA1) and refraction (sphere SPH1, cylinder CYL1, axis AX1, and spherical equivalent SE1). Uncorrected distance (UDVA2) and corrected distance visual acuity (CDVA2) and refraction (sphere SPH2, cylinder CYL2, axis AX2, and spherical equivalent SE2) after surgery were also registered. SE is defined as sphere plus half the cylinder. The refractive prediction error (PE), defined as the postoperative spherical equivalent, minus the predicted refraction, was calculated. Demographic data, such as age and sex, were likewise documented. Ethnicity is not registered in Cuban protocols, so its influence cannot be analyzed [17].

Axial length was divided in four groups: short (<22 mm), medium (22–23.99 mm), long (24–26 mm), and very long (>26 mm) eyes. There are reported different criteria for myopia and hyperopia [10, 18]. In this paper, we classified as myopic those eyes with a refractive spherical value under – 0.50 diopters (D) and hyperopic eyes when these values were over + 0.50 D. Astigmatism was classified as “with-the-rule” (wtr) when the steep meridian on the corneal surface was in the range 60–120°, or “against-the-rule” (atr) when the steep meridian was 0–30° or 150–180°. The remaining cases of astigmatism were classified as oblique. Keratometric astigmatism is defined as the difference of SK–FK.

The dependence of the number of patients having a given cylinder with the cylinder value (the distribution of cylinder values) is used to fit an exponential model. The model is

(1) $$\begin{array}{c} y=A\;\exp\left\{-\frac{x}{\chi }\right\}. \end{array}$$

In Equation (1), *x* is the cylinder in diopters; *A* is the pre-exponential factor, which depends on the size of the sample; *y* is the number of patients with the cylinder in a given interval with *x* in the center; *e* (2.71828..) is the base of the natural logarithms; and *χ* is the decaying factor, which depends on how fast the number of patients with a given cylinder decreases with the increase of it. The larger the *χ*, the slower the decrease of *y* is. In this sense, *χ* characterizes the distribution of the magnitude considered.

The distribution of implanted IOL and refractive errors were also analyzed, obviously excluding from the sample the patients who became aphakic.

In patients with both eyes measured, refractive anisometropia was considered as SE differences ≥1.0 D between eyes. We also considered the anisometropia in the sphere. The distribution of the number of eyes with a given magnitude of the difference is used to fit the same exponential model of Equation (1). The influence of AL and ACD in anisometropia is also studied.

The distribution of ocular biometric characteristics, visual acuity, and refractive results were analyzed both across the entire study population and segmented by sex (males and females) and age (<40, 40–59, 60–80, and >80 years old).

Statistical analyses were performed using the free software R, v. 3.6.3. The results are expressed as the parameter mean (with a confidence interval of 95%), median, standard deviation (SD), variance (Var), skewness, kurtosis, and the interquartile range (IQR), defined as the difference between the third quartile and the first one *Q*_3_–*Q*_1_. Skewness (*γ*) is a measure of the asymmetry of the distribution of the considered random variable around its mean. A normal distribution (and every symmetric distribution) has zero skewness. A negative skew (left-skewed) indicates that the left tail is more important in the distribution than the right one, while a positive skew (right-skewed) indicates the contrary. Kurtosis (*κ*) is a measure of the tailedness of the distribution of the random variable; it is related with the probability of production of outliers according to the distribution. It is commonly compared with the kurtosis of a normal distribution (3) saying that a distribution is platykurtic if its kurtosis is smaller than three and leptokurtic if it is larger. The difference between the kurtosis of a given distribution and the normal kurtosis is named excess kurtosis. All these magnitudes characterize the random variable distribution.

Differences between two levels of a variable were assessed for significance using the Student's *t*-test for unpaired samples, given as *t* and *p* values. Those values of *p* < 0.05 were considered statistically significant. The difference between several levels is elucidated with an analysis of variance test. These criteria were used for age- and sex-related differences.

A Kolmogorov–Smirnov goodness of fit test was used to determine if the studied parameters were normally distributed or not.

## 3. Results and Discussion

### 3.1. Results

Table 1 shows the selected magnitudes and their units, as well as the number of eyes that were included and excluded from the results for each one. Note that the number of eyes used in the analysis of the different magnitudes changes. The reasons for the differences are diverse:

- The measurement was not registered in the database
- IOL Master 3.0.2 and 500 does not measure the lens thickness
- The patient became aphakic
- Preoperative refraction was not completed for unknown reasons
- Patients did not attend postoperative refraction appointment

Though the number of patients with registered LT is much less than the total number of eyes, this number is large enough to yield sound statistics. The statistical distribution of the involved magnitudes was assessed calculating their momenta and related values: mean, median, standard deviation (*σ*), skewness, and kurtosis. In Table 2, the values of momenta and related quantities for the different random variables considered are shown. In Table S1 of Supplementary Material, the 95% confidence intervals of the magnitudes (expressed as the mean $\pm 1.96\sigma /\sqrt{n}$ , *σ* the standard deviation, and *n* the number of patients) and the Interquartile Range are presented. The Kolmogorov–Smirnov test showed that none of the variables is normally distributed.

The segmentation by age groups is shown in Figure S1 (Supplementary Materials).  Figure S1(a) shows a histogram of the distribution of age, in intervals of five years. Average age is 68.70 years, with a standard deviation of 10.89 years. The distribution is left skewed, as expected, and platykurtic, with a kurtosis close to three.  Figure S1(b) shows the distribution among age groups. The larger percent is, as usual, in the age group between 60 and 80 years.

**Figure 1:**
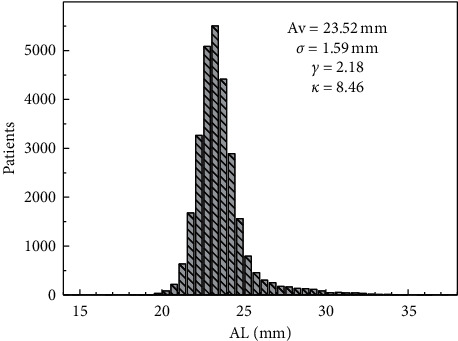
Histogram of the axial length, with 0.5 mm bins. In the inset, the main statigraphs of the distribution are shown.

Figure 1 shows the AL's distribution. It has two interesting characteristics. Firstly, it is right-skewed, meaning that the right tail is longer. In the second place, it is highly leptokurtic, with an excess kurtosis of 5.46, meaning that the distribution has fat tails. The combination of these two characteristics indicates that there are more extra-long eyes (AL > 30 mm, 272 eyes) than extra-short ones (AL < 20 mm, 36 eyes). The average AL is 23.52 mm (minimum 14.98 mm, maximum 37.40 mm, *σ* = 1.59 mm).  Figure S2 shows the axial length distribution of the sample, segmented by length groups. Note the predominant group is in the normal length range.

**Figure 2:**
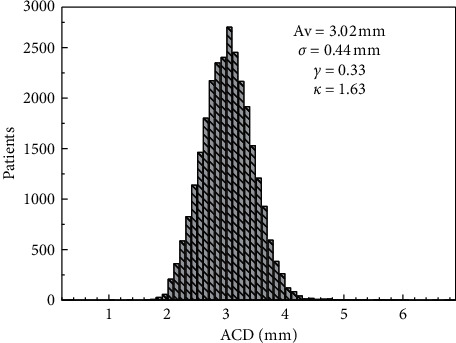
Histogram of the anterior chamber depth with bins of 0.2 mm. In the inset, the main statigraphs of the distribution are shown.

Figure 2 presents the distribution of ACD. Average ACD is 3.02 mm (*σ* = 0.44 mm), with a small skewness (0.33) and a small kurtosis (1.63), which is reflected in the thin tails that can be appreciated from the figure.  Figure S3 shows a histogram of LT values. The crystalline thickness has an average value of 4.55 mm (*σ* = 0.52 mm). It is possible to see that the distribution is highly symmetric (*γ*  ≈ 0) and slightly leptokurtic (*κ*  ≈ 3).

**Figure 3:**
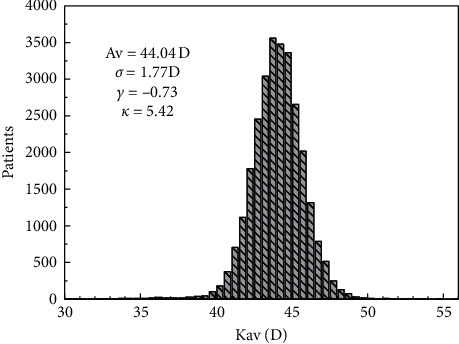
Histogram of the average corneal power with 0.5 D bins. In the inset, the main statigraphs of the distribution are shown.

A histogram of Kav is shown in Figure 3. Its average value is 44.04 D (*σ* = 1.77 D). The distribution of values of SK and FK is shown in Figures S4(a) and S4(b), respectively. The average values are 44.61 (*σ* = 1.86 D) and 43.47 D (*σ* = 1.80 D).

**Figure 4:**
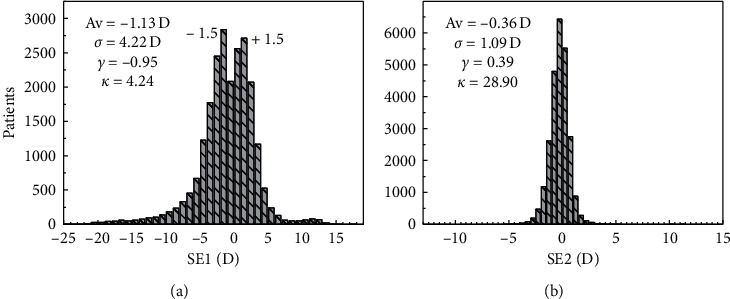
Histograms of SE, with 1 D bins. (a) Preoperative, it is possible to note a bimodal distribution. (b) Postoperative. Comparing both figures, note the effect of the surgical procedure. In the insets, the main statigraphs of the distributions are shown.

The preoperative SE has a bimodal distribution, as shown in Figure 4(a). The two modes are -1.50 D (myopic) and +1.50 D (hypermetropic). The average preoperative SE is −1.13 D with a standard deviation of 4.22 D.  Figure S5(a) is a histogram representing the distribution of the preoperative sphere. It is evident that the preoperative sphere is also bimodal (modes −1.5 D and +1.5 D, average of −0.52 D), influencing the distribution of the SE. The distribution of the SE after surgery is different, as can be seen in Figure 4(b). In this case, the distribution is unimodal, with an average value of −0.36 D (*σ* = 1.09 D).

Figure S5(b) is the histogram of SPH2 that has an average value of 0.41 D (*σ* = 1.06 D).

The distribution of cylinders has a maximum in 0.75 D and decreases exponentially (accordingly with Equation (1)) as the cylinder increases, as can be seen for CYL1 in Figure S6(a). The decay rate is *χ* = 0.92 D, with an Adjusted *R*^2^ = 0.9995. The decrease of CYL2 is also exponential (see Figure S6(b)), decaying slower (decay rate of 1.36 D) and *R*^2^ = 0.9945.

**Figure 5:**
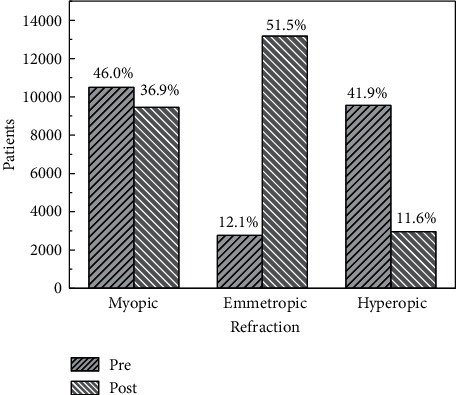
Classification of the refraction, preoperative and postoperative. Note that the total number of patients in the preoperative and postoperative stages is different (see Table 1).

Figure 5 shows the distribution of eyes classified as myopic, emmetropic, and hyperopic, before and after surgery. The distribution of refractive defects changes after surgery. The relative proportion and the absolute number of emmetropic eyes increase notably. The number and percent of hyperopic and myopic eyes considerably decreases. Note that the number of eyes measured after surgery is larger than before (see Table 1).

**Figure 6:**
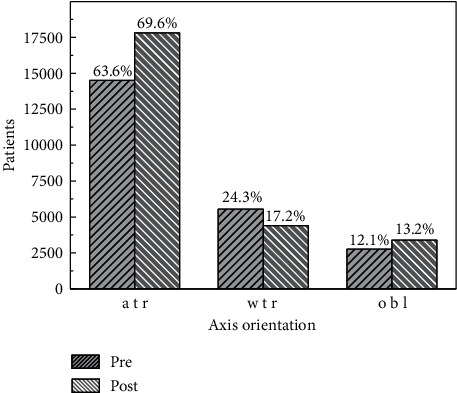
Proportion of type of astigmatism, preoperative and postoperative. wtr: with the rule; atr: against the rule; obl: oblique. Note that the total number of patients, preoperative and postoperative, are different (see Table 1).

Regarding the type of astigmatism, Figure 6 indicates that the number and percent of eyes showing atr astigmatism increases after the surgery. The number and proportion of wtr astigmatism diminish while the number and the proportion of eyes with oblique astigmatism increase slightly after surgery. Note that the number of eyes used for determining AX1 and AX2 is different (see Table 1).

**Figure 7:**
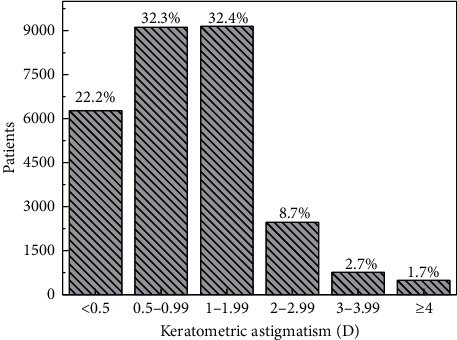
Histogram of the keratometric astigmatism.

Keratometric astigmatism has a distribution shown in Figure 7. This histogram tells us that more than half of the patients have keratometric astigmatism below 1 D. The average value is 1.15 D, with a standard deviation of 0.96 D. The distribution is right-skewed, and highly leptokurtic.  Figure S7 shows that the distribution of keratometric astigmatism has an exponential shape, with a decaying factor of 0.858 D.

**Figure 8:**
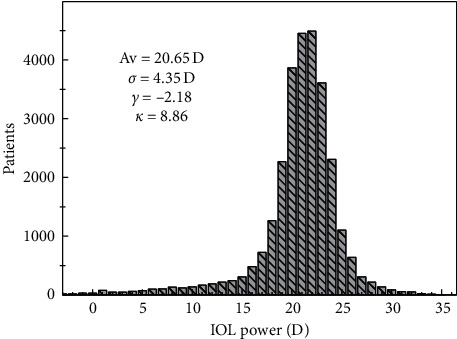
Histogram of the implanted lens power, with 1 D bin. In the inset, the values of the main statigraphs are shown.

**Figure 9:**
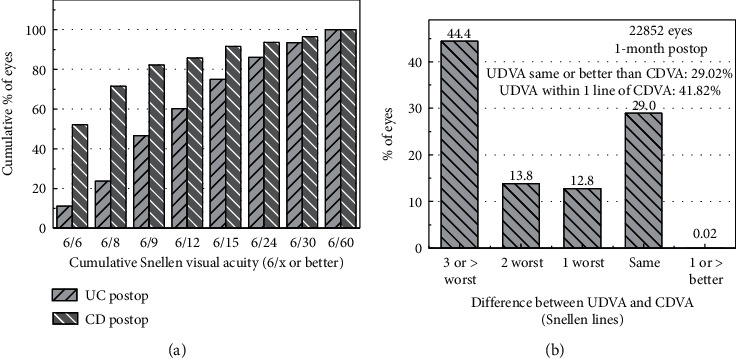
Distribution of the postoperative visual acuity (both uncorrected distance UD and corrected distance CD). (a) The percent of eyes with a given visual acuity. (b) Differences between UD and CD in terms of Snellen lines.

Figure 8 shows the distribution of IOL of the lenses implanted to the patients. It is easy to see that the more frequently implanted lenses have a power between 20 D and 22 D (12759 lenses, 45.3%).  Figure S8 shows a histogram of uncorrected distance (UD) and corrected distance (CD) visual acuity before and after surgery. Before surgery, most of the patients have an uncorrected visual acuity below 0.3, while the corrected visual acuity is below 0.6 in a majority of the cases. After surgery, on the contrary, 47% of the patients have uncorrected distance visual acuity above 0.5, and 64% have corrected distance visual acuity above 0.8. As the cataract has a major influence on visual acuity, the postoperative results are more important. In Figure 9 the postoperative uncorrected and corrected distance visual acuity are compared.  Figure 9(a) compares the cumulative percent of both quantities, while Figure 9(b) shows the difference between them in terms of Snellen lines.

**Figure 10:**
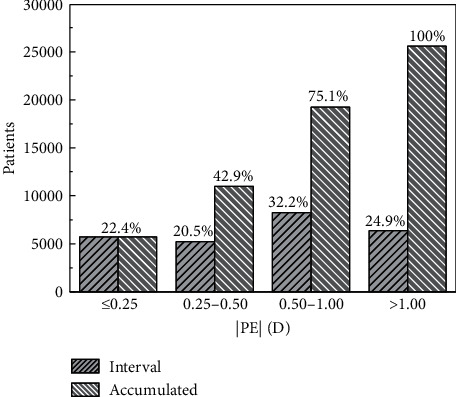
Distribution of the absolute value of the PE in intervals, and its cumulative values.

Figure 10 shows the distribution of the absolute value of PE in different intervals. In the figure, both the number and percent of eyes in each interval are presented. Around 75% of eyes reach a postoperative refraction that differs less than one diopter of the predicted one.  Figure S9 shows the distribution of PE, with a mean value of 0.28 D (*σ* = 0.99 D). The presence of refractive surprises is signaled by the high value of kurtosis. This magnitude and its dependence with the formula used to calculate the prediction refraction (including the optimization of the constants used), as well as the lens model, are so important that they deserve a separate study. It will be conducted in a forthcoming paper.  Figure S10 shows the distribution of anisometropia before surgery.  Figure S10(a) represents the distribution of the absolute value of the difference SPH1_OS_-SPH1_OD_ and the fit of an exponential model (Equation (1)) to the experimental data.  Figure S10(b) is similar, but the magnitude is related to the absolute value of the difference SE1_OS_-SE1_OD_. Overall, of the 1863 pair of eyes having preoperative data, 1038 (55.7%) present anisometropia of the sphere, while 959 (51.5%) present anisometropia of the spherical equivalent. Both distributions have an exponential decay (*R*^2^ > 0.99), with similar decaying factors (1.16 and 1.11 D, respectively).

The effects of the surgery on the anisometropia are evident by analyzing Figure S11.  Figure S11(a) shows the distribution of the SPH2's anisometropia and Figure S11(b) shows the distribution of the anisometropia in SE2. Of the 2632 measured pairs of eyes, 636 (24.2%) show anisometropia in the sphere and 773 (29.4%) in the spherical equivalent. Not only the proportion of affected eyes diminishes but also the prevalence of large anisometropias decreases. It is evident in the values of the decaying factor (0.71 and 0.620 D, respectively).

An additional study of the anisometropia in the biometric parameters of the mate eyes was performed. The difference |AL_OS_–AL_OD_| has an average value of 0.24 mm (*σ* = 0.45 mm, IQR = 0.17 mm). When the same calculation is performed with the ACD, |ACD_OS_–ACD_OD_| has an average value of 0.18 mm, *σ* = 0.32 mm, and IQR = 0.17 mm.

Table 3 reports some statigraphs of different magnitudes segmented by sex. Female eyes are shorter and have shallower anterior chambers than male eyes. The average LT of both sexes are statistically different, with female lenses thicker than the male ones. These factors combined imply that men have deeper vitreous chambers than females. The power of the female's corneas is consistently bigger than that of males. It is so not only for the average power but also for the extreme keratometries.

The average value of SE1 is more myopic in male patients than in female ones. After surgery, though the differences between the average SE of males and females diminish, the values are significantly different. CYL1 and CYL2 are different between sexes, though in the post-stage the differences between the distributions are less important.

Consistent with the biometric findings, female eyes need in average more powerful implanted lenses than men. The standard deviation, skewness, and kurtosis of the distributions segmented by sex are most of times very close, implying that both distributions are only displaced, having similar shapes.

**Figure 11:**
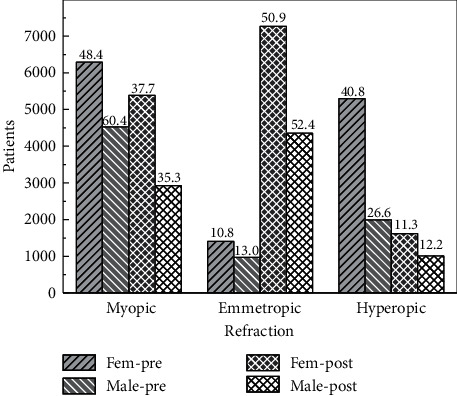
Classification of the refraction, preoperative and postoperative, segmented by sex.

Figure 11 presents the percent distribution of refraction, before and after surgery, segmented by sex. The distribution before surgery shows that for males, the majority of eyes (60%) are myopic, while for females the prevalence of myopia is larger than the prevalence of hyperopia, but the difference is not as large. Both sexes present a small number of emmetropic eyes, slightly above 10%. After surgery, the proportion of emmetropic eyes increases up to 50%, diminishing the percent differences among sexes in the three segments. Regarding anisometropia, both sexes present the same prevalence (*p* = 0.45).

Table 4 reports the segmentation by age of the biometric magnitudes, as well as the refraction (pre- and post-) and PE for the selected age groups. A one-way ANOVA test was performed to detect differences between mean values of the different groups. The bottom row of the table gives the F-values obtained, as well as the real power of the test. The values per age group of AL, LT, ACD, and SE1 present highly significant differences between all groups.  Table 5 shows the behavior of the average keratometry. The age group of 41–60 presents differences with all other groups that do not present differences among them. PE, on the contrary, has a different behavior, explained in Table 6. SE2 does not present differences among age groups.

Note that in all these magnitudes, the standard deviation and other statigraphs (not shown here) change between groups.

### 3.2. Discussion

The results of the present hospital-based population study constitute, to our knowledge, the largest and more representative investigation on ocular biometric parameters of Cuban cataract patients using optical biometry. In addition, extensive data on refractive errors and visual outcomes distribution are also provided by this work.

Missing information of some variables in numerous cases, due to aforementioned dissimilar reasons, makes it mandatory to exclude certain amount of analyzed eyes for specific parameters. Since available data of each magnitude is enormous, these exclusions did not hinder from obtaining representative results of every one of them.

Mean age in our study (68.70 years) is consistent with the frequent cataract association to senescence and its left-skewed distribution, showing that the larger percent in the age group between 60 and 80 years behaves similar as vastly reported on literature in all latitudes [19, 20]. Most previous Cuban studies [13, 15, 21] agree with our finding. The average age in Ref. [22] is smaller than the value found in this study. It also happens in Ref [16], dedicated to study young hyperopes patients. Ref. [14] does not report age data.

The standard value of AL of the human eye is internationally taken to be around 24 mm in adulthood regardless of sex or race, whereas AL tends to be longer in myopic and shorter in hypermetropic eyes comparing to that of emmetropic [23]. The right-skewed distribution is in accordance with the predominance of myopia in the studied sample. AL average values are variables in the literature. The mean AL observed in this paper is larger than those of Refs. [19, 22, 24], in which ultrasound, low-coherence reflectometry, and partial coherence interferometry are used, respectively. Conversely, Refs. [2, 25] using low-coherence reflectometry and partial coherence interferometry, respectively, show larger average values. Previous researches in Cuba report larger [26], much larger [15], shorter [27], and much shorter [16] average AL when compared with our results. It should be noted that the analyzed periods in all these works include only one year or less, and that the sample size is much smaller than ours, explaining the dispersion in their results. Specifically, in the case of Ref. [15], more than 93% of patients had myopic astigmatism while the result of Ref. [16] is in accordance with his study´s design, where all the patients were hyperopes.

Average ACD in our work (3.02 mm) is shallower than reported in Latino populations [11] and to a lesser extent, those of Refs. [28, 29] in Asian populations. On the other hand, Hashemi et al. [24] report an ACD average even shallower than ours using the biometer LENSTAR/BioGraph (WaveLight AG, Erlangen, Germany). Differences between these two biometers may affect the comparative analysis since ACD value in Lenstar refers to the distance between corneal endothelium and lens anterior capsule while the distance for this variable in IOL Master is taken from corneal epithelium to lens anterior capsule. That is why Lenstar uses the sum of central pachymetry and ACD values for IOL calculation [27]. Therefore, this issue has to be considered when comparing Lenstar and IOL Master as Cuban researchers did in a previous study [27], showing values closer to ours, with no significant differences between these two biometers. In contrast, Song et al. [4] observed that ACD measured by IOL Master 500 was shallower than that measured by IOL Master 700 or Lenstar LS900 (Haag Steit AG, Koeniz, Switzerland)). In Song's study, the ACD mean value is similar to ours when measured by IOL Master 500, while values from IOL Master 700 and Lenstar are deeper. This result corresponds with that of the number of eyes measured using PCI (IOL Master 3.0.2 and 500) in our sample is much bigger than those measured by SS-OCT. A former study conducted in the Cuban Institute of Ophthalmology [26], using optic (IOL Master 500) and immersion ultrasonic biometry, shows similar results to ours. We found no ACD mean values reported in other Cuban population-based studies.

Accommodation must be considered for an accurate measurement of lens thickness. Nonstandardized accommodative stimuli used for research and the wide resolution variability among the available techniques to measure this magnitude explain the difference in lens thickness reported in literature. However, many studies, including this, are conducted without recording accommodative response, assuming that the subject was accommodating accurately to the target [30, 31]. In addition, considering those patients under 40 years old in the present study, the nonpresbyope ones represent only 1.45% of the sample and all patients had been diagnosed with cataract, making improbably that lens thickness would be significantly affected by accommodation. Since PCI-based biometers are not able to offer this magnitude, lens thickness was measured in 4395 eyes by using exclusively the SS-OCT-based IOL Master 700, which offers the best resolution, approximately 10–20 *μ*m.

The average LT of 4.55 mm is higher than that reported in studies that used an A-scan ultrasound, which has lower resolution than the SS-OCT, for studying Latino [11, 19] and elderly Chinese populations [10]. Hashemi et al. [24] and Ferreira et al. [2], using both low-coherence optical reflectometry (Lenstar) observed much smaller LT values. As far as we know, a previous report of this magnitude has not been published on Cuban patients.

Keratometry average value and its distribution in our study are close to those observed in European population-based studies using partial-coherence interferometry [20] and optical low-coherence reflectometry [2].

In the Asian population, Song et al. [4] show similar keratometry average value when using Lenstar and IOL Master 700 and a bit bigger when using IOL Master 500, while Wickremasinghe et al. [18] show much close values to ours, using an autorefractor–keratometer (Canon RK-5 Autorefractor Keratometer; Canon, Inc., Tokyo, Japan). Cuban researchers [15, 32] using autorefractor–keratometers have previously reported slightly lower values. Keratometry measurements may vary when different evaluation methods are used, as it was demonstrated in Ref. [33] reporting a significantly lower corneal power when measurements were obtained with autorefractor–keratometers than those measured by IOL Master.

Relating to FK and SK, the average values are 43.47 D and 44.62 D, respectively. The Auckland Cataract Study [34], in a defined New Zealand population, found a near value for average flattest keratometry and a rather lower value for the steepest one. Likewise, in a preceding Cuban study [27], FK and SK mean values were lower than ours, using both Lenstar and IOL Master 500.

The bimodal distribution of SPH1, with both myopic and hypermetropic peaks, has a notorious influence in the similar bimodal behavior of SE1 distribution. SE1 shows a myopic mean value. Though correlation between myopia and cataract is not a purpose of this paper, this myopic SE1 may be related in some extent to the presence of nuclear cataract, taking into account the previously reported association between these variables. [35].

Similar myopic SE1 is reported in Ref. [36]. In Refs. [15, 22], the spherical component shows a similar influence on the prevalence of ametropias in Cuban samples. SE2 mean value within emmetropia range (±0.5 D) is the expected outcome and it is similar to those reported in literature [34].

Before analyzing astigmatism behavior, it is important to remark that keratometry is not commonly measured following cataract surgery at the postoperative stage in Cuba, unless it is required for investigative purposes. Therefore, only refractive astigmatism is available after surgery in this retrospective study. In our series, both corneal and refractive average astigmatism is larger than 1 D before surgery. However, taking into account that most of total astigmatism comes from the cornea, and lenticular astigmatism is eliminated with extraction of the cataract, only the corneal astigmatism is considered when planning cataract surgery.

The distribution of CYL1 (see Figure S6(a)) for values above 1 D is exponential. In the inset of this figure, the fit of an exponential model to the experimental values is shown. The high value of *R*^2^ (0.9995) indicates that the model explains almost all the variations observed. Below this value, a marked decrement in the number of patients is found. Attebo et al. [37] found a similar behavior, though they do not make a fitting of a model to the data. They also found a decrement in the number of cases for values of cylinder below 0.5 D. As they note, it may be associated with a preference of not to report these small cylinders, as participants could usually read the same number of letters without it.

A similar dependence is found for CYL2 (Figure S6(b)). The exponential model explains more than 99% of the variation of the cylinder. The main difference between both distributions is the value of the decaying factor *χ*. While for CYL1 it is 0.92 D, for CYL2 it is 1.36 D, indicating a slower decrement. This is a signal that, as a result of the incision, the refractive astigmatism increases. This is confirmed by the average values of both magnitudes, reported in Table 2.

Keratometric astigmatism behaves similar to that of the preoperatory refractive one; in both cases, almost 90% of the eyes have a value below 2 D.  Figure S7, represents the distribution of the keratometric astigmatism. The exponential fitting shown in the inset has a similar decrement as that presented in Figure S6(a). Note that the decaying factor of the keratometric astigmatism (0.858 D) is close to the decaying factor of the preoperative refractive astigmatism (0.92 D), indicating the close relation between both magnitudes.

The increment of astigmatism after surgery may be explained by the lack of toric lenses and the nonsystematic practice of anti-astigmatic incisions by the surgeons due to nonavailability of graduated scalpels in our environment. At the Cuban Institute of Ophthalmology, most of the surgeons habitually perform the principal corneal incision during phacoemulsification on temporal or supero-temporal sites, since incisions on these locations are less astigmatogenic. Some others treat astigmatism up to 1.5 D by making the principal corneal incision on the steepest meridian during cataract surgery. In some cases, this may be technically difficult and may require less comfortable positioning and consequently it is frequently avoided by surgeons having a negative impact on postoperative astigmatism. In a study conducted in the United Kingdom, where nontoric standard monofocal IOLs were implanted in 99 percent of the eyes, a similar trend toward greater postoperative astigmatism was observed [38]. Despite the nondisposable toric IOL or graduated scalpels for limbal releasing incision (LRI), a former Cuban study showed a mean surgical induced astigmatism (SIA) of 0.61 D after phacoemulsification [39], whereas SIA resulted in higher than 1 D when superior sclera-corneal incisions were performed [40].

ATR-astigmatism represents, by far, the major percentage at preoperative stage. Similar behavior has been observed in Refs. [2, 5, 41] and previous Cuban researches [15, 22].

The preoperative refractive defect with a higher prevalence is myopia. Since the strong correlation between axial length and ocular refraction have been formerly confirmed [42], the aforementioned presence of more extra-long eyes than extra-short ones observed in this study's sample may be a contributing factor for this fact. In addition, the probably larger lens thickness related to the condition of cataract diagnosis for all patients selected in this study, with respect to the noncataractous population, may be related to an increased lens refractive power. Olsen et al. [42] observed that the stronger the lens, the more myopic the eye. Although the present paper did not explicitly analyze the lens' refractive power, this magnitude is significantly associated with higher lens thickness [43]. The result of the present study is coincident with the predominance of myopic eyes reported in the preoperative setting by Fernandez et al. [15] and Santos et al. [21] in former Cuban studies.

At the postoperative stage, the percent of emmetropic eyes increases notably leading to an improvement in both UDVA and CDVA. This is the expected outcome considering that the purpose of modern cataract surgery is not limited to remove the opacified crystalline lens but to offer the best visual and refractive outcomes without the use of spectacles. Comparing with previous Cuban studies, our results are better than that reported by Galindo Zamora et al. [14]. Conversely, Santos et al. [21] show a major percentage of eyes with better UDAV and CDVA than the present paper, but the studied sample was quite smaller than ours. These good visual outcomes are much related with refractive PE distribution showing a postoperative refraction that differs less than one diopter of the predicted one. The average PE is smaller than those of Refs. [44–46]. However, those authors found better results than ours when analyzing the percentage of eyes with average refractive prediction error within ±0.50 D. The high excess of kurtosis of this parameter can explain this behavior, and it is in accordance with the almost 25% of refractive surprises observed in this paper. A previous Cuban study shows a smaller percentage of eyes with average PE in a range inferior to 0.50 D, using third-generation formulas in patients who underwent uneventful cataract surgery from 2007 to 2011 [47].

Anisometropia, in both SPH and SE, is present in more than 50% of patients whose preoperative data of both eyes were registered. This value is quite higher than those reported in previous Cuban studies [15, 22]. It must be noted that in those studies, anisometropia was considered when the difference between the spherical components of both eyes was higher than 2.5 D, which explains the discordance with respect to our results. However, Akhgary et al. [48] analyzing patients in a wide range of age (1–80 years old) observed that anisometropia was higher than in aforementioned Cuban studies but still pretty smaller than ours.

The high percentage of anisometropia observed in this paper may be related to the predominance of patients beyond 60 years old (more than 80%). The marked increment in the prevalence of anisometropic refractive errors later in life has been demonstrated [49]. An extensive review about anisometropia [50] concluded that, although asymmetric cataract development or unilateral cataract extraction has been suggested as a cause of anisometropia in older adults, the more probable origin for this ametropia in senescence may be related to a regression of neural control of binocular vision. The exponential decay in its distribution shows that the prevalence of large differences between both eyes before surgery (decaying factor around 1.1 D) is alleviated by surgery, giving a smaller decaying factor (≤0.71 D). Average value of the difference |AL_OS_–AL_OD_| is significantly bigger in patients with anisometropia than in those who do not suffer from this refractive disturbance, while the average of difference |ACD_OS_–ACD_OD_| is not. This result suggests a positive correlation between the unequal axial length growth of both eyes and anisometropia. Coincident results have been observed in a study that examined the prevalence of anisometropia and interocular differences of ocular biometric parameters among elderly female twins, showing that higher anisometropia concerning both spherical refraction and spherical equivalent were associated with more myopic refraction and longer AL [51]. Unlike the present study, some authors [52, 53] have found a positive correlation between the anterior chamber depth difference and anisometropia, specifically in hyperopic anisometropic eyes. The predominance of myopia in our sample may explain the noncoincident findings concerning ACD, since no division was made between anisomyopes and anisohyperopes.

When comparing different magnitudes segmented by sex, females have shorter eyes, shallower ACD, and steeper corneas. Coincident results have been reported in several studies in Asian [9, 18, 24, 54], European [2], and Latino [11, 19] populations. However, Lee et al. [25] found that the association of sex with AL and ACD were no longer significant after adjustment for height, suggesting that the usually smaller stature of women may be the real cause of smaller eyes. Many other studies have reported the association between height and AL [55]. One study [56] stated that sex-differences with respect to AL and ACD were still significant in multivariate analyses controlling for stature, suggesting that sex may be an independent determinant of AL. Likewise, in Ref. [2], it was concluded that the greatest predictor of ocular biometrics is gender. However, these gender differences related to AL are not significant in emmetropic subjects as reported by another study [23]. An average flatter cornea observed in males is similar to reports in literature but, unlike AL and ACD, it seems to be not influenced by height adjustments [25].

Lens thickness is significantly bigger in females. Other authors have reported similar results [19, 54]. In contrast, another study [57] found that males had slightly greater thicknesses than females but the mean difference was not significant. In the Los Angeles Latino Eye Study [11], a significant gender-related difference in lens thickness was found, although it was not statistically significant after adjusting for height.

The abovementioned findings are the reason for the greater average value of the IOL power implanted to females.

Refractive error is probably a consequence of a mismatch of the biometric parameters of the eyes (i.e. AL, K, ACD, LT) and the refractive power of these structures [53, 58]. AL (or vitreous chamber depth when AL is broken down into its components) has been remarked as the most important contributor parameter in determining refractive error, followed by the corneal power of the eye [11]. These authors also found that other variables had a smaller but significant contributing effect on refractive error such as lens opalescence and the two other components of axial length: lens thickness and anterior chamber depth. Correspondingly, the deeper anterior chamber and vitreous chamber and congruently the longer AL may explain the preponderance of preoperative myopia in males, considering both the distribution and the average spherical equivalent. Other studies in the Chinese population report that women tend to be more hyperopic [58, 59]. However, in the present paper, myopia also exhibits higher prevalence than hyperopia in females, but the difference is not as large as that observed in males. On the other hand, other studies found nonsignificant sex difference in refractive error [10].

Because all of the aforementioned biometric parameters for each individual are considered for IOL calculation in order to achieve a better visual and refractive outcome, the percent differences of myopia and hyperopia between sexes diminish while emmetropia increases after cataract surgery. The average SE of both males and females is closer to zero, and the difference between them diminishes after surgery, although the values remain significantly different. The relevance of these gender differences in ocular biometry has been considered in formulating some of the newer generation IOL calculation formulas [60, 61]. Anisometropia has not been associated with gender as an independent factor. Inter-gender difference of anisometropia is not significant in this study coinciding with previous reports in the Cuban population [15, 22].

Biometric parameters show significant differences when age is considered. The younger the patients the longer AL and deeper the ACD, while LT tends to be thicker in older patients. One study analyzing 750 cataractous eyes in an US population [62] showed similar correlations. Analogous results are also reported in Iranian [24], Australian [57], and Los Angeles Latino populations [11] for these three magnitudes. However, another study [10] found that AL did not change with age although ACD showed a decrease. Keratometry particularly shows no difference among ages, except for the 41–60 group, which significantly differs from the other groups. This result is different from that reported in Ref. [57], where no significant change was observed for anterior corneal radii of curvature related to age. He et al. [10] observed that corneal power did not change with age either.

Regarding refractive error and age, SE1 shows a shift to hyperopia as patients get older. This outcome is coincident with the refraction patterns described in Ref. [63] in which refraction is stable from 20 s to 40 s and then moves in the hypermetropic direction. Likewise, a former study in Cuba [34] found that ametropia with a hypermetropic component was more frequent in the older age groups, while those with a myopic component predominated in the younger age groups. A study in a Latino population [11] shows a coincident result. In Ref. [10], the spherical equivalent tended to become hyperopic at 60 years and shifted toward myopia at 75 years. Similarly, the Blue Mountains Eye Study [63] observed that a myopic shift in refraction occurred most frequently in older (≥70 years) than younger (<70 years) participants. Another study [44] shows that in the 50–59 year age group and the 60–69 year age group, there was a hyperopic shift of 0.41 D and 0.34 D, respectively.

## 4. Conclusions

In summary, this paper describes the distribution of ocular biometric characteristics, visual acuity, and refractive results in a large sample of the Cuban population.

The preoperative biometric parameters show highly significant differences between sexes and age, with male and younger eyes being longer and with a deeper anterior chamber, but with a thinner lens. Keratometry (KS, KF, and Kav) shows highly significant sex differences, being steeper in female eyes than in males; however, age-related differences are not significant except for the 41–60 group. Before surgery, both sexes have myopic eyes on average, with male eyes being more myopic than female eyes. Preoperative spherical equivalent tends to hyperopia with age. After surgery, the average spherical equivalent is −0.36 D, and female eyes are more myopic than males (*p* < 0.001). The percent of emmetropic eyes increases notably, leading to a significant improvement in both UDVA and CDVA, evidencing the tremendous impact of cataract surgery in reducing preoperative ametropias.

These results are of interest not only in Cuba but also in regions with a large Cuban and Cuban descendant population, such as several places in the USA (particularly the south of Florida), Spain, and several counties of Latin America. This would also be important in other countries with similar ethnic composition (a mixing of Europeans from the Iberian Peninsula and Black Africans) in the Caribbean region and South America.

## Figures and Tables

**Table 1:** Definition of the used variables, their units, and the amount of eyes included and excluded.

Variables	Def (units)	Included	Excluded
AGE*∗*	Age (years)	25068	0
AL	Axial length (mm)	28240	12
ACD	Anterior chamber depth (mm)	27923	229
LT	Lens thickness (mm)	4395	23857
SPH1	Sphere of the preoperative refraction (D)	22818	5434
CYL1	Cylinder of the preoperative refraction (D)	22818	5434
AX1	Preoperative axis (degree)	22818	5434
SE1	Preoperative spherical equivalent (D)	22818	5434
SK	Power of the steepest meridian (D)	28248	4
FK	Power of the flattest meridian (D)	28248	4
Kav	Average corneal power (D)	28248	4
UDVA1	Preoperative uncorrected distance visual acuity	28252	0
CDVA1	Preoperative corrected distance visual acuity	28252	0
IOL	IOL power (D)	28252	0
SPH2	Sphere of the postoperative refraction (D)	25589	2663
CYL2	Cylinder of the postoperative refraction (D)	25589	2663
AX2	Postoperative axis (degrees)	25589	2663
SE2	Postoperative spherical equivalent (D)	25589	2663
UDVA2	Postoperative uncorrected visual acuity	28252	0
CDVA2	Postoperative best corrected visual acuity	28252	0
PE	Prediction Error (D)	25589	2663

*∗*In the AGE field, only the number of patients is considered.

**Table 2:** Statistical parameters of the considered variables.

Variables	Mean	Median	SD	Var	Skewness	Kurtosis
AGE (years)	68.70	70.00	10.89	118.61	−1.06	2.32
AL (mm)	23.52	23.28	1.59	2.52	2.18	8.46
ACD (mm)	3.02	3.02	0.44	0.19	0.33	1.63
LT (mm)	4.55	4.55	0.52	0.27	−0.24	3.41
SPH1 (D)	−0.52	−0.25	4.17	17.37	−0.90	4.27
CYL1 (D)	1.22	1.00	0.90	0.81	1.48	4.04
AX1 (deg)	82.78	85.00	43.96	1932.54	−0.02	0.02
SE1 (D)	−1.13	−0.88	4.22	17.84	−0.95	4.24
SK (D)	44.61	44.64	1.86	3.47	−0.53	5.83
FK (D)	43.47	43.54	1.80	3.23	−0.84	5.32
Kav (D)	44.04	44.07	1.77	3.12	−0.73	5.42
UDVA1	0.16	0.10	0.14	0.02	1.83	3.98
CDVA1	0.40	0.40	0.27	0.07	0.54	-0.41
IOL (D)	20.65	21.00	4.35	18.89	−2.18	8.86
SPH2 (D)	0.41	0.50	1.06	1.13	0.72	22.54
CYL2 (D)	1.52	1.25	1.08	1.17	1.40	2.98
AX2 (deg)	93.62	95.00	38.50	1482.39	−0.27	0.66
SE2 (D)	−0.36	−0.25	1.09	1.03	0.39	28.90
UDVA2	0.46	0.40	0.28	0.08	0.39	−0.81
CDVA2	0.77	0.90	0.28	0.08	−1.11	0.16
PE (D)	0.28	0.34	0.99	0.98	−0.06	19.44

**Table 3:** Gender segmentation of the data.

Variables	Sex	Mean	Median	SD	Var	Skew	Kurt	Number	*t*-test	*p* value
Age	F	68.77	70.00	10.70	114.49	−0.94	1.81	17811	**0.78**	**0.43**
	M	68.67	70.00	10.93	119.51	−1.24	3.09	10441		

AL	F	23.31	23.02	1.63	2.67	2.35	8.66	17802	30.91	<0.001
	M	23.88	23.72	1.43	2.05	2.30	10.62	10437		

ACD	F	2.95	2.95	0.42	0.18	0.33	1.40	17628	31.66	<0.001
	M	3.13	3.13	0.44	0.20	0.31	2.22	10294		

LT	F	4.57	4.58	0.50	0.25	−0.23	2.07	2681	2.58	0.01
	M	4.53	4.50	0.55	0.31	−0.22	4.70	1714		

SK	F	44.87	44.88	1.83	3.36	−0.60	6.02	17807	30.91	<0.001
	M	44.17	44.18	1.83	3.34	−0.48	6.73	10440		

FK	F	43.74	43.77	1.77	3.15	−0.96	6.52	17807	34.31	<0.001
	M	43.00	43.05	1.74	3.03	−0.77	4.66	10440		

Kav	F	44.31	44.33	1.74	3.03	−0.81	5.83	17807	33.83	<0.001
	M	43.59	43.64	1.72	2.94	−0.73	6.08	10440		

SE1	F	−0.91	−0.38	4.36	19.03	−1.14	4.26	12989	10.05	<0.001
	M	−1.52	−1.50	4.03	16.22	−0.61	4.39	7498		

SE2	F	−0.37	−0.38	1.03	1.06	0.36	29.66	14269	3.45	<0.001
	M	−0.32	−0.25	1.02	1.03	0.21	28.28	8296		

CYL1	F	−1.18	−1.00	0.89	0.79	−1.61	4.78	12989	7.07	<0.001
	M	−1.27	−1.00	0.92	0.85	−1.37	3.43	7498		

CYL2	F	−1.49	−1.25	1.07	1.16	−1.45	3.21	14269	3.36	<0.001
	M	−1.54	−1.25	1.09	1.19	−1.40	3.15	8296		

IOL	F	21.04	22.00	4.57	20.88	−2.23	8.18	17810	20.62	<0.001
	M	19.99	20.00	3.85	14.80	−2.32	11.99	10441		

PE	F	0.27	0.32	1.03	1.06	−0.32	27.16	14269	**1.75**	**0.08**
	M	0.29	0.34	1.00	0.99	0.14	19.93	8296		

A Student's *t* test for unpaired samples was performed in order to compare the means of each variable among sexes. *p*–value < 0.05 is considered as highly significant. Nonsignificant values are shown in boldface.

**Table 4:** Segmentation by age of the biometric parameters, spherical equivalents (SE1 and SE2), and PE.

Age group	AL (mm) mean (sd)	LT (mm) mean (sd)	ACD (mm) mean (sd)	Kav (D) mean (sd)	SEpre (D) mean (sd)	SEpost (D) mean (sd)	PE (D) mean (sd)
≤40	24.61 (2.99)	3.87 (0.58)	3.39 (0.55)	44.17 (2.37)	−3.43 (8.54)	−0.44 (1.35)	0.13 (1.53)
41–60	23.95 (2.45)	4.31 (0.52)	3.18 (0.45)	43.83 (2.18)	−2.05 (6.11)	−0.37 (1.26)	0.32 (1.36)
61–79	23.44 (1.32)	4.58 (0.50)	3.00 (0.42)	44.08 (1.66)	−1.03 (3.56)	−0.33 (1.03)	0.28 (1.07)
≥80	23.28 (0.99)	4.73 (0.52)	2.85 (0.41)	44.13 (1.56)	0.06 (2.78)	−0.35 (1.18)	0.20 (1.77)
F	204.2 (1)	95.9 (1)	497.8 (1)	Table 5	392 (1)	**No diff. (1)**	Table 6

The results of the analysis of variance are reported using the *F*-value, with the actual power of the test in parentheses. The analysis of Kav and PE is particularized in Tables 5 and 6, respectively.

**Table 5:** Kav.

	41–60	61–79	≥80

≤40	*p* < 0.001	**No (p** = **0.32)**	**No (** *p* = **0.72)**
41–60		*p* < 0.001	*p* < 0.001
61–79			**No (** *p* = **0.12)**

**Table 6:** Prediction error.

	41–60	61–79	≥80

≤40	*p* < 0.05	*p* < 0.05	**No (** *p* = **0.45)**
41–60		*p* < 0.001	*p* < 0.001
61–79			**No (** *p* = **0.18)**

## Data Availability

The data are available upon request to the corresponding author.

## Supplementary Materials

Supplementary Materials Table S1: 95% confidence intervals and interquartile range of the selected magnitudes. Figure S1: age distribution of the sample: (a) Histogram with a bin size of five years, showing in the inset the main statigraphs of the distribution. (b) Age groups and the percent of eyes belonging to each one. Figure S2: number of eyes in the different length ranges: short (<22 mm), medium (22–23.9 mm), large (24–26 mm), and very large (>26 mm). The percent of eyes in each group is presented. Figure S3: histogram of the lens thickness, with 0.2 mm bin size. In the inset, the main statigraphs of the distribution are shown. Figure S4: histogram of the keratometries with 0.5 D bins. (a) Steepest keratometry. (b) Flattest keratometry. In the insets, the main statigraphs of both distributions are shown. Figure S5: histograms of the sphere of the refraction with 0.5 D bins: (a) Preoperative sphere. (b) Postoperative sphere. In the insets, the main statigraphs of both distributions are shown. Figure S6: histograms of the refractive cylinder with 0.5 D bins. (a) Preoperative. (b) Postoperative. In the insets, the fitting of an exponential model (Equation (1)) to the data is shown. The value of the decaying factor *χ* is given with its standard error in parentheses. Figure S7: keratometric astigmatism, the distribution function and the fit of an exponential model to the data. The value of the decaying factor *χ* is given with its standard error in parentheses. Figure S8: visual acuity, uncorrected distance (UD), and corrected (CD), before and after surgery. Figure S9: distribution of PE. In the inset, some statigraphs of the distribution are shown. Figure S10: anisometropy in the preoperative stage. (a) In the sphere. (b) In the spherical equivalent. In the insets of both graphs, the results of the fitting of an exponential model (Equation (1)) to the data are shown. The decaying factor is presented with its standard error in parentheses. Figure S11: anisometropy in the postoperative stage. (a) In the sphere. (b) In the spherical equivalent. In the insets of both graphs, the results of the fitting of an exponential model (Equation (1)) to the data are shown. The decaying factor is presented with its standard error in parentheses.
