# Supplementary material for: Biometry, Refractive Errors, and the Results of Cataract Surgery: A Large Sample Study
Source: J Ophthalmol. 2021 Apr 27;2021:9918763. doi: 10.1155/2021/9918763 (PMC8099509; doi:10.1155/2021/9918763)
Supplement: Supplementary Materials — Table S1: 95% confidence intervals and interquartile range of the selected magnitudes. Figure S1: age distribution of the sample: (a) Histogram with a bin size of five years, showing in the inset the main statigraphs of the distribution. (b) Age groups and the percent of eyes belonging to each one. Figure S2: number of eyes in the different length ranges: short (<22 mm), medium (22–23.9 mm), large (24–26 mm), and very large (>26 mm). The percent of eyes in each group is presented. Figure S3: histogram of the lens thickness, with 0.2 mm bin size. In the inset, the main statigraphs of the distribution are shown. Figure S4: histogram of the keratometries with 0.5 D bins. (a) Steepest keratometry. (b) Flattest keratometry. In the insets, the main statigraphs of both distributions are shown. Figure S5: histograms of the sphere of the refraction with 0.5 D bins: (a) Preoperative sphere. (b) Postoperative sphere. In the insets, the main statigraphs of both distributions are shown. Figure S6: histograms of the refractive cylinder with 0.5 D bins. (a) Preoperative. (b) Postoperative. In the insets, the fitting of an exponential model (Equation (1)) to the data is shown. The value of the decaying factor χ is given with its standard error in parentheses. Figure S7: keratometric astigmatism, the distribution function and the fit of an exponential model to the data. The value of the decaying factor χ is given with its standard error in parentheses. Figure S8: visual acuity, uncorrected distance (UD), and corrected (CD), before and after surgery. Figure S9: distribution of PE. In the inset, some statigraphs of the distribution are shown. Figure S10: anisometropy in the preoperative stage. (a) In the sphere. (b) In the spherical equivalent. In the insets of both graphs, the results of the fitting of an exponential model (Equation (1)) to the data are shown. The decaying factor is presented with its standard error in parentheses. Figure S11: anisometropy in the postoperative stage. [file 9918763.f1.docx]

Table S1: 95% Confidence Intervals and inter-quartile range of the selected magnitudes.

| Variable | Conf. Int 95% | | 1st Quart | 3rd Quart | Int. Quart. |
| --- | --- | --- | --- | --- | --- |
| Age (y) | 68,560 | 68,830 | 63,00 | 76,00 | 13,00 |
| AL (mm) | 23,502 | 23,539 | 22,62 | 24,03 | 1,41 |
| ACD (mm) | 3,012 | 3,022 | 2,72 | 3,30 | 0,58 |
| LT (mm) | 4,536 | 4,567 | 4,23 | 4.87 | 0,64 |
| SPH1 (D) | -0,572 | -0,464 | -2,5 | 2 | 4,5 |
| CYL1 (D) | 1,204 | 1,228 | 0,50 | 1,50 | 1,00 |
| AX1 (dg) | 82,208 | 83,349 | 65,00 | 105,00 | 40,00 |
| SE1 (D) | -1,181 | -1,071 | -3,00 | 1,50 | 4,50 |
| SK (D) | 44,592 | 44,636 | 43,53 | 45,73 | 2,20 |
| FK (D) | 43,448 | 43,490 | 42,45 | 44,58 | 2,13 |
| K (D) | 44,021 | 44,062 | 43,01 | 45,13 | 2,13 |
| UDVA1 | 0,161 | 0,165 | 0,08 | 0,20 | 0,12 |
| CDVA1 | 0,398 | 0,404 | 0,20 | 0,60 | 0,40 |
| IOL (D) | 20,596 | 20,698 | 20,00 | 23,00 | 3,00 |
| SPH2 (D) | 0,393 | 0,419 | 0,00 | 1,00 | 1,00 |
| CYL2 (D) | 1,511 | 1,538 | 0,75 | 2,00 | 1,25 |
| AX2 (dg) | 93,151 | 94,095 | 80,00 | 110,00 | 30,00 |
| SE2 (D) | -0,369 | -0,344 | -0,88 | 0,25 | 1,13 |
| UDVA2 | 0,453 | 0,459 | 0,20 | 0,60 | 0.40 |
| CDVA2 | 0,764 | 0,771 | 0,60 | 1,00 | 0.40 |
| PredErr (D) | 0,272 | 0,297 | -0,21 | 0,82 | 1,02 |





Figure S1: Age distribution of the sample: a) Histogram with a bin size of five years, showing in the inset the main statigraphs of the distribution. b) age groups and the percent of eyes belonging to each one.





Figure S2: Number of eyes in the different length ranges: short (< 22 mm), medium (22 – 23.9 mm), large (24 – 26 mm) and very large (> 26 mm). The percent of eyes in each group in presented.





Figure S3: Histogram of the lens thickness, with 0.2 mm bin size. In the inset the main statigraphs of the distribution are shown.





Figure S4: Histogram of the keratometries with 0.5 D bins. a) Steepest keratometry. b) Flattest keratometry. In the insets, the main statigraphs of both distributions are shown.


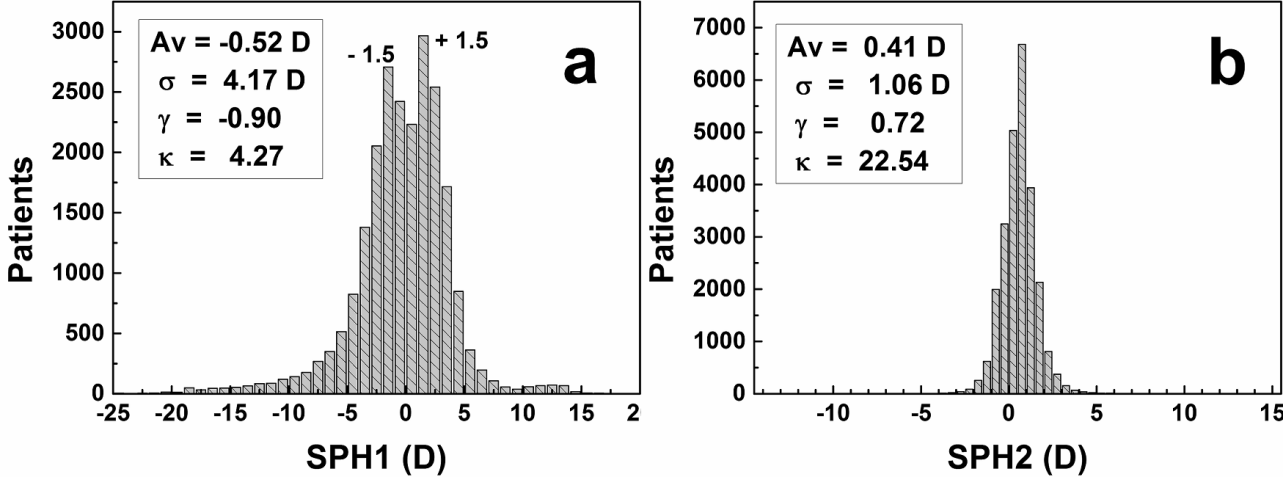


Figure S5: Histograms of the sphere of the refraction with 0.5 D bins: a) Preoperative sphere. b) Postoperative sphere. In the insets, the main statigraphs of both distributions are shown.


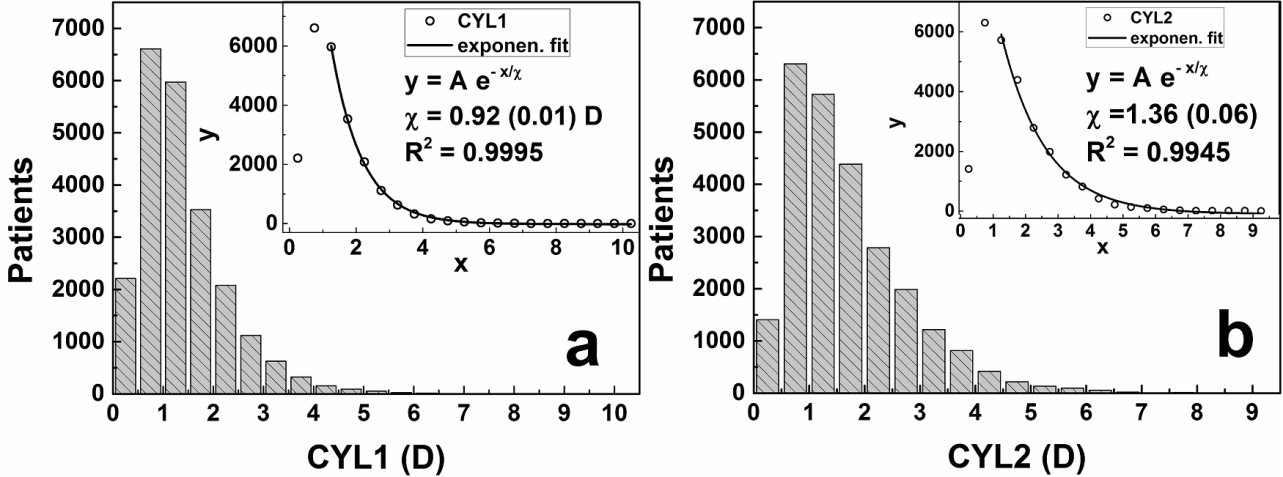


Figure S6: Histograms of the refractive cylinder with 0.5 D bins. a) Preoperative. b) Postoperative. In the insets, the fitting of an exponential model (Eq. (1)) to the data is shown. The value of the decaying factor *χ* is given with its standard error in parentheses





Figure S7: Keratometric astigmatism, the distribution function and the fit of an exponential model to the data. The value of the decaying factor *χ* is given with its standard error in parentheses.





Figure S8: Visual acuity, uncorrected distance (UD) and corrected (CD), before and after surgery.





Figure S9: Distribution of PE. In the inset, some statigraphs of the distribution are shown.


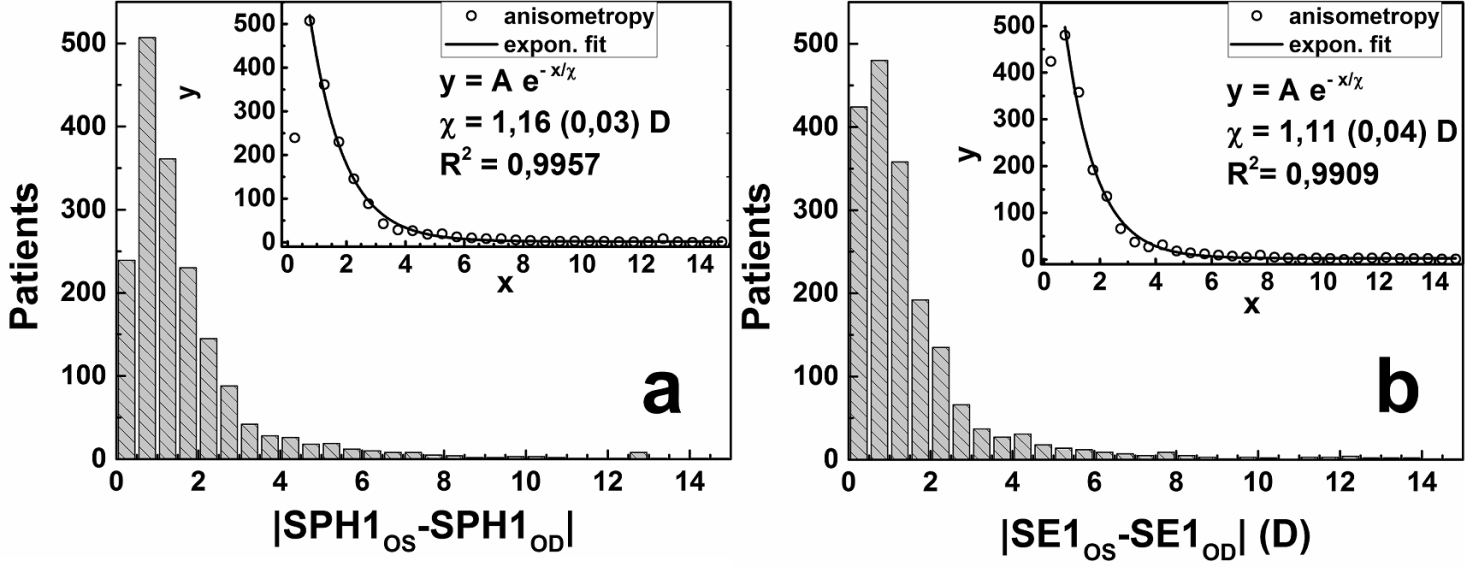


Figure S10: Anisometropy in the preoperative stage. a) in the sphere, b) in the spherical equivalent. In the insets of both graphs, the results of the fitting of an exponential model (Eq. (1)) to the data are shown. The decaying factor is presented with its standard error in parentheses.


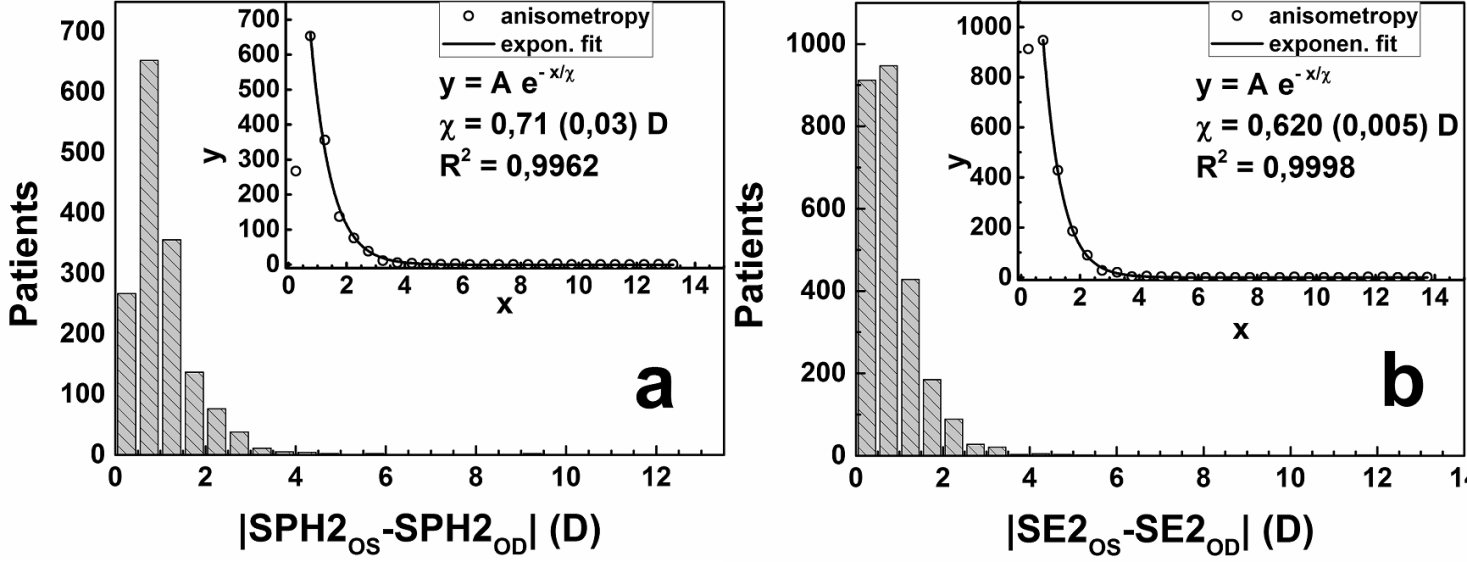


Figure S11: Anisometropy in the postoperative stage. a) in the sphere, b) in the spherical equivalent. In the insets of both graphs, the results of the fitting of an exponential model (Eq. (1)) to the data are shown. The decaying factor is presented with its standard error in parentheses.
